# Advances in mass spectrometry-based clinical biomarker discovery

**DOI:** 10.1186/s12014-015-9102-9

**Published:** 2016-01-07

**Authors:** Christopher A. Crutchfield, Stefani N. Thomas, Lori J. Sokoll, Daniel W. Chan

**Affiliations:** Department of Pathology, School of Medicine, Johns Hopkins University, Baltimore, MD 21287 USA; Department of Pathology and Laboratory Medicine, College of Medicine, University of Cincinnati, Cincinnati, OH 45219 USA

**Keywords:** Mass spectrometry, Biomarker, Proteomics, Clinical performance

## Abstract

The greatest unmet needs in biomarker discovery are those discoveries that lead to the development of clinical diagnostic tests. These clinical diagnostic tests can provide early intervention when a patient would present otherwise healthy (e.g., cancer or cardiovascular disease) and aid clinical decision making with improved clinical outcomes. The past two decades have seen significant technological improvements in the analytical capabilities of mass spectrometers. Mass spectrometers are unique in that they can directly analyze any biological molecule susceptible to ionization. The biological studies of human metabolites and proteins using contemporary mass spectrometry technology (metabolomics and proteomics, respectively) has been ongoing for over a decade. Some of these studies have resulted in exciting insights into human biology. However, relatively few biomarkers have been translated into clinical tests. This review will discuss some key technological developments that have occurred over this time with an emphasis on technologies that will create new avenues for biomarker discovery.

## Background

Laboratory medicine has a tremendous impact on clinical decision making. Currently, most routine chemistry tests utilize spectrophotometric or immunologic detection schema. Mass spectrometry (MS) typically provides much greater analytical specificity relative to these methods. Mass spectrometry has been used routinely in the clinical laboratory, primarily in the context of toxicological testing and therapeutic drug monitoring. The success of mass spectrometry in these clinical testing arenas is juxtaposed by a promise of a golden age of biomarker discovery. This review will present a contemporary perspective on the challenges and opportunities for biomarker discovery as well as speculate on their future clinical applications. It will also address how technical innovation has instilled new promise for mass spectrometry based biomarkers, including both protein-based and small molecule-based biomarkers. Finally, it will present the foundational terminology necessary for evaluating biomarkers in a clinical context.

## The clinical need for biomarkers

The majority of clinical decisions are based on laboratory test results. Practice guidelines from professional societies optimized clinical decisions that influence clinical outcomes, particularly in the interpretation of testing related to endocrine function, cancer markers, or cardiac markers [1, 2]. As an example, markers of cardiovascular health have gained considerable utility in the past three decades as published in 2014 by the American College of Cardiology (ACC) [2]. With the utilization of cardiac troponin for detection of myocardial infarction and B-type natriuretic peptides for detection of congestive heart failure, the clinic has very specific and sensitive tests to evaluate cardiovascular status using laboratory tests. However, even as successful as troponin has been in ruling out myocardial infarction, its diagnostic sensitivity and specificity are not “perfect,” and with chest pain being the second most common reason for emergency room visits, there is a need for further investment in discovering better markers [3]. Moreover, laboratory tests that are used in other areas of medicine, such as those intended for cancer screening, still require a considerable amount of development and validation before they can be approved by the FDA and used clinically [4].

Notably, there is a significant distinction between biomarker *discovery* and biomarker *validation*. As will be noted later in the manuscript there are several examples of promising biomarkers. Due to the statistical nature of sampling hundreds or thousands of biological components, many experiments will have a “significant” feature that is suggestive of a “true biomarker.” Much of the criticism of biomarker discovery focuses on the rift between biomarker discovery and biomarker validation—where a validated marker has a defined clinical utility demonstrated across multiple patient populations. While the discovery component is challenging and expensive (requiring expensive equipment, trained personnel, and precious specimens) the clinical validation component can be even more challenging, with coordinating the implementation of a technology across many geographic locations and recruiting many patients to test biomarker robustness. Biomarker validation is outside the scope of this review, which will focus principally on new technical avenues for biomarkers that may have greater clinical promise than those already in the validation pipeline [5].

## Clinical utility of biomarkers

Laboratory medicine has been practiced for centuries. Over the past century, however, technology has enabled novel perspectives on human health and disease by measuring the chemical composition of human body fluids. A language of laboratory medicine has been developed that describes performance characteristics and applications of biomarkers. Biomarker utilization in the clinic depends on diagnostic sensitivity and specificity for evaluating a condition, and in the US, biomarker utilization is contingent upon FDA evaluation for a particular “Intended Use”. Generally, a biomarker’s clinical performance is designated by its diagnostic sensitivity and diagnostic specificity. Diagnostic sensitivity is the likelihood that the diagnostic test will return a positive test result when testing a patient with the disease. Diagnostic specificity is the likelihood that the diagnostic test will return a negative result when testing a patient that does not have the disease. The sensitivity or specificity of a test is a direct result of the “cutoff” level of the test. Another name for this level is the medical decision point. In biomarker applications that produce a qualitative result, sensitivity, specificity, or a combination of the two may be maximized. When used for screening, tumor markers frequently have high diagnostic sensitivity so as to not miss any cancers, however specificity is important to avoid additional, and potentially costly, follow-up testing.

In qualitative urine toxicology testing, positive immunoassay screening results that often focus on broad drug classes are frequently confirmed using mass spectrometry. This is a consequence of the exquisite analytical *specificity* provided by mass spectrometry (the ability of the mass spectrometer to discriminate between different molecules). Another example to illustrate this point is the measurement of 25 (OH) vitamin D in the clinic. Vitamin D is measured on automated immunoassay platforms using a binding assay that does not discriminate between vitamin D2 and vitamin D3, whereas mass spectrometry based methods can discriminate between the two forms [6]. LC–MS/MS provides a multitude of analytical parameters that may be adjusted to enhance the analytical selectivity of an analyte. Principally these are the chromatographic conditions (of which there are many, with regards to both the solid phase and liquid phase components of the analyzer), the ionization conditions (ionization method, polarity, and source conditions), and finally the mass spectrometer itself (choosing appropriate parent and fragment *m*/*z* transitions to monitor with commiserate collision energies and mass accuracy). This exquisite analytical specificity causes issues during biomarker discovery when analyzing digested proteins because protein digestion may homogenize a protein isoform that has high diagnostic performance in such a way as to strip it of its diagnostic specificity (for example, a protein might have high diagnostic performance if it has three phosphorylated residues but low diagnostic performance if it has zero, one, or two. Upon digestion the total number of phosphorylation events can be lost). Consequently, the vast majority of clinical laboratory testing relies on non-MS based detection methods. One major driving factor of the clinical laboratory’s non-reliance on mass spectrometry is that the majority of clinical laboratory tests have already been FDA approved on automated platforms which have the capacity to analyze a large menu of tests. Another force driving the lack of utilization of MS-based methods is the relatively high capital acquisition costs of a MS, costs of training personnel to operate the analyzers, and the relatively low test throughput compared to automated chemistry analyzers.

There is a plethora of potential applications of biomarkers in the clinic, namely

- Screening/diagnosis/detection
- Prognosis and prediction
- Monitoring

### Screening/diagnosis/detection

Screening tests, when successful, are generally very beneficial to clinical outcomes. For example, early detection of cancer provides opportunities to remove malignant tissue before it metastasizes to other organ systems. In 2000, the National Cancer Institute (NCI) established an initiative titled the Early Detection Research Network (EDRN) which has as its objective facilitating the development of biomarkers or technology that enable early detection of cancer. It achieves this main objective through funding, evaluating markers and technology, enabling the collaboration of academic and industrial leaders of cross-disciplinary fields, and by disseminating the results [7].

### Prognosis and prediction

Some biomarkers provide prognostic information about disease outcome. For example, while not mass-spectrometry-based, gene expression has tremendous prognostic information when evaluating patients with breast cancer. Patients with “triple-negative” breast cancer (not expressing ER, PR, or HER2) have significantly lower survival rates than other breast cancers [8]. Predictive markers are used to select and assess targeted therapies.

### Monitoring

Biomarkers are also frequently monitored throughout the course of a patient’s disease. One marker that has enabled alternative surgical approaches to hyperparathyroidism has been intraoperative PTH [9]. Quantitative application of this marker enables minimally invasive surgery for parathyroid resection. After the surgeon has removed the hyperfunctioning tissue, a concomitant decrease of PTH levels by >50 % suggests full resection. Most FDA cleared tumor markers are for monitoring of therapies (e.g., CA 125 for ovarian cancer and CA 27.29 for breast cancer).

## Classification of biomarkers

### Protein biomarkers

Protein biomarkers represent a significant number of all markers used for routine care in the clinic. For example, albumin can be used as a nutritional marker, alanine aminotransferase (ALT) can be used as a marker for liver dysfunction, and fecal elastase can be used as a marker of pancreatic insufficiency. However, the clinical assays for all of these markers do not require mass spectrometry. Dialogue regarding the successes or failures of proteomics needs to be held within the framework that non-mass spectrometry-based analytical methodologies have already had relatively great success at providing clinical insight in patient pathophysiology and they provide improved clinical outcomes when well utilized. Nonetheless, the majority of the proteins used for routine clinical care diagnoses are relatively high abundance (especially albumin). The monolithic challenge in developing a new protein biomarker assay is developing one that not only has the requisite mass spectrometric sensitivity (with the appropriate dynamic range) but that could also be adopted in the clinic in a way to either justify the expense of a mass spectrometer *or* enable conversion to a more cost efficient technology (e.g., spectrophotometric or immunologic). As protein biomarkers are already routinely utilized in the clinic using standard analytical techniques, the opportunity for the clinical application of mass spectrometry is to find the analytical niches it can *solely* provide access to. These applications will be made possible primarily through the exquisite analytical specificity mass spectrometry provides that immunologic or spectrophotometric-based methods cannot achieve. These applications will likely *not* be “protein” based, but rather utilize the investigation of *post*-*translational modifications* of proteins, the presence or concentration of small molecule metabolites, or profiling metabolic flux.

### Biomarkers with protein post-translational modification

Routine clinical assays of proteins use many different methodologies for analysis. Despite the methodology they often disregard specific protein isoforms and frequently present protein concentration as the sum of all isoforms. This convention disregards the *explicit* post-translational modification state of the protein or enzyme. Herein lays the “holy grail” of clinical proteomics: identifying molecularly specific isoforms of proteins that provide unparalleled clinical sensitivity and specificity.

There are over a dozen distinct post-translational modifications that can modulate protein signaling or enzyme activity [10]. It is possible that all will eventually find a niche. Presently, the most commonly studied PTMs are

- Glycosylation
- Methylation, acetylation and ubiquitination
- Phosphorylation

#### Glycosylation

Glycosylation is one of the most complex protein modifications. It is also one of the most promising protein modifications for new biomarker development because of recent advances in the technology required for its investigation. Glycosylated proteins have one or more oligosaccharides attached to a Ser/Thr (O-linked glycosylation) or an Asn residue (N-linked glycosylation) [11]. The molecular signaling that dictates the stoichiometry and coordination of sugar branching is not fully understood. It is clear, however, that the glycan patterns observed in cancerous cells can be distinct in different cell types, such as core-fucosylation [12]. A practical example is the improvement of detecting aggressive prostate cancer using serum fucosylated prostate specific antigen (PSA). Serum fucosylated prostate-specific antigen (PSA) improves the differentiation of aggressive from non-aggressive prostate cancers [13].

The primary technical challenges in addressing glycosylation-based biomarkers are:

- Glycan heterogeneity: Most mass analyzers do not have the sensitivity to adequately determine the “micro”-heterogeneity of protein glycosylation.
- Enrichments of glycoproteins: Compounding the previously mentioned issue of glycan heterogeneity, the primary method for analysis of glycoproteins uses glycoprotein enrichment technologies [14].
- Glycan sequencing algorithms: Even when glycan components of glycoproteins can be isolated, the computational challenges in constructing an accurate glycan structure from the mass spectra are problematic. Most methods utilize databases and scoring systems, but these methods inherently bias the investigator to known glycans (rather than possibly novel glycoforms) [15].

#### Methylation, acetylation and ubiquitination

While methylation, acetylation, and ubiquitination have a role in many protein classes, major interest has developed in their role in the deciphering of the human “histone code.” Histones are proteins that package DNA in nucleosomal units that ultimately form chromosomes. These proteins are subjected to a variety of post-translational modifications, including methylation, acetylation, phosphorylation, and ubiquitination [16, 17]. These modifications directly modify protein expression. It is thought that disease-associated pathology can be treated in cells with aberrant protein expression (e.g., cancer) by targeting “mis-coded” histones or applying “histone modification therapy” [18]. Although these strategies have seen some use in clinical research studies, they have not been as consistently beneficial as in pre-clinical models [19].

#### Phosphorylation

The majority of intracellular molecular signaling pathways rely on phosphorylation events. The development of mass spectrometry-based detection of phosphorylation events has been high risk due to technical difficulty, but may result in a new class of biomarkers. Due to their intrinsic importance in signaling, they have high potential for communicating pathological states, but on the other hand, due to their high energy bonds, they are less stable and thus more prone to analytical artifacts [20]. Some strategies for isolating phosphopeptides include immobilized metal affinity chromatography, reversible covalent binding, metal oxide affinity chromatography, and magnetic beads [21, 22].

### Metabolic biomarkers

While most contemporary discussions of new biomarkers have focused on proteins and post-translational modifications, new opportunities are arising from the improved characterization of the impact of disease on human small molecule metabolite concentrations and flux. One of the most highly cited metabolic consequences of disease is the “Warburg effect,” [23] whereby cancer cells utilize an accelerated rate of glycolysis for energy production even in highly aerobic conditions. Understanding this physiologic behavior has enabled scanning technologies such as positron emission tomography, which uses a labeled form of deoxyglucose, which cannot be further metabolized but will be taken up by cancer cells at a faster rate than healthy cells, to localize cancer in a patient’s body. With this understanding, even though there have only been limited discoveries in the production of small molecule metabolite-based biomarkers of cancer, the technology has set the stage for more discoveries in the near future.

One of the most promising demonstrations of the power of small molecule mass spectrometry in advancing our understanding of cancer, and providing an opportunity for a mass spectrometry-based small molecule metabolite biomarker, is the discovery of (*R*)-2-hydroxyglutate and its interaction with isocitrate dehydrogenase mutations [24–26]. Mutations in this enzyme alter its catalytic activity and result in the production of the *oncometabolite* (*R*)-2-hydroxygularate (R-2-HG), which is normally produced at very low levels in healthy cells. The presence of R-2-HG has been speculated to promote transformation of healthy cells into cancers through a variety of mechanisms that are outside the scope of this review. However, the discovery of R-2-HG demonstrates the power of current MS-based detection methods for biomarkers. Principally, extracts of cultured glioma cells expressing either WT or mutant IDH were profiled using LC–MS, coupling reverse phase chromatography to a standalone Orbitrap mass spectrometer scanning in negative ionization mode in the *m*/z range of 110–1000 Da with a resolution of ~100,000. With the raw data generated, “untargeted” profiling requires the generation of a feature map, which attempts to assimilate all the mass spectra in an experiment into “features,” which represent co-eluting ion species. These ion species will include the ionized form of monoisotopic metabolites (typically, uniformly 12-C and 14-N, as well as their heavy forms due to the natural isotopic abundance +1, or +2 Da, in addition to ionization adducts with other salts, such as Na+).

#### Kinetic flux profiling

Mass spectrometry technology will create opportunities for novel strategies that combine developments in organic synthesis, biological sampling strategies, and complex mass spectrometry analysis. With advances in fast scanning triple quadrupole mass spectrometers as well as high resolution mass spectrometers, technology is available to quantify the flux of the glycolytic pathway as well as other degradation pathways that branch off of it. A theoretical testing strategy would be a “challenge” for an oncology patient with a difficult to reach tumor with a suspected metabolic subtype. Assuming the cancer has accelerated metabolic rates relative to basal metabolism, an isotopically labeled metabolic tracer (for example, labeled glucose or glutamine) could indicate the highly active metabolic pathways in the cancer. A drug targeting those highly active metabolic pathways may improve the treatment of the patient. This future view of laboratory medicine improves on the paradigm of knowing where the tracer is localizing by providing information regarding its fate after being metabolized. This technology is just beginning to mature, but is principally limited to cell cultures. It has been applied to better identify targets for antiviral therapy [27] as well as attempt to investigate chemotherapeutic mechanisms, such as methotrexate in the treatment of breast cancer [28].

## Mass spectrometry technologies

### Mass spectrometers

Triple quadrupole mass spectrometers are most commonly found in clinical labs for quantitative analysis. These instruments achieve analytical specificity through multiple analytical stages. A quadrupole itself is an orientation of metal rods that filter mass ions by alternating current, creating a stable oscillation (and hence, transmission) of a “band” of a selected *m/z* ions. The precision of this selection is typically ~1 Da. The “triple quadrupole” refers to the series of quadrupoles oriented in a way that selects for *m/z* twice (the 1st and 3rd quadrupole), where the 2nd quadrupole fragments the ions selected from the first filtering stage. This fragmentation stage provides additional analytical specificity. While many ionized molecules (and adducts) may share nominally identical parent *m/z*, they produce unique fragmentation products. The third quadrupole takes advantage of this fact by selecting specific fragmentation products that are generated. The terminology used to describe this type of ion detection depends specifically on the quantity of mass transitions monitored: selected reaction monitoring (SRM) or multiple reaction monitoring (MRM). SRM detection only monitors for a single transition during an analysis. MRM detection monitors for a series of transitions during an analysis (though only one at any given time). While most triple quadrupole mass spectrometry has been applied for small molecule analysis, in recent years, there has been a movement to perform MRM-based analysis of peptide products of protein digests [29]. This strategy has benefitted from the development of software that facilitates the selection of transitions for detecting a peptide [30]. Moreover, triple quadrupole mass spectrometers can also be used to screen for biomarkers wherein the strategy for achieving analytical specificity takes advantages of the process of unique fragmentation [31–33]. These experiments are primarily performed using a scanning mode called “precursor ion scan.” In this scanning mode, the triple quadrupole holds the last mass filter constant (for a particular product ion resulting from a particular collision energy) and scans a range of parent *m/z* values for those that produce the desired fragment. This strategy can be useful when targeting a known class of compounds that produce identical fragments (phosphates, sulfates, steroids, etc.). The MRM assay could be used to screen a large number (several hundreds) of potential biomarkers in a multiplex fashion. The result could be used to select a smaller set of promising biomarkers for further validation, most likely by immunoassays. The approach of using MRM MS has the advantage of being faster (with multiplexing) and less expensive (without the need for antibody development).

Hybrid instruments typically refer to high resolution instruments coupled to a front-end component that enables fragmentation (Q-ToF, Triple-TOF, Q-Orbitrap). These analyzers are ubiquitous with biomarker discovery studies because of their unparalleled analytical specificity. Compared to a triple quadrupole analyzer, however, they may have less analytical sensitivity (less capacity to detect a low concentration analyte). From a biomarker discovery perspective, the advantage of hybrid mass spectrometers results from their capacity to scan a chromatographic analysis for highly mass resolved analytical features that are significant via absolute or relative quantification and their ability to then provide additional structural information for either a triggered or retrospective fragmentation event. This analytical strategy is employed for either small molecule screening, or for shotgun proteomics (where proteins are digested to peptides and sequenced by their MS/MS spectra). The Triple-TOF, due in part to its lower duty cycle compared to an Orbitrap-based analyzer, has also enabled a detection schema called sequential windowed acquisition of all theoretical ions (SWATH), which attempts to analyze the fragmentation products of all ions generated during an analysis, otherwise known as data independent acquisition [34–36]. Other approaches for data independent acquisition exist, and are generally limited to the vendor of the mass spectrometer [37, 38].

### Ionization sources

Electrospray (ESI) revolutionized biological mass spectrometry because it provided a conventional method by which biologically derived molecules could be conveniently transitioned from the liquid phase (necessary for liquid chromatography) to the gas phase (necessary for mass spectrometry) [39]. Before its inception, most attempts at biological mass spectrometry were performed using GC–MS, which required chemical derivatization for most molecules. The discovery of ESI was so profound that the scientist who discovered it, John Fenn, was awarded the Nobel Prize in Chemistry in 2002 (the award was shared with Koicihi Tanaka for the discovery of matrix-assisted laser desorption ionization, MALDI) [40]. Other technical options exist for interfacing a liquid phase molecule to the gas phase, principally atmospheric pressure chemical ionization and atmospheric pressure photoionization [41], though these ionization techniques are typically relegated to more niche classes of compounds (more nonpolar than most biomolecules).

Matrix-assisted laser desorption ionization (MALDI) has the benefit of low pre-analysis work-up. The typical workflow involves mixing samples with a chemical matrix followed by the generation of ions with the application of a high energy laser. This is a very convenient strategy for analyzing simple protein digests (peptide mass fingerprinting [42]). However, as the complexity of the specimen (or number of chromatography fractions) increases, the duration of pre-analysis starts to become longer than that of simply coupling a liquid chromatograph system to an electrospray ionization source. A niche in which MALDI has provided tremendous in-roads in clinical utility has been the microbiology laboratory. MALDI is quickly becoming the *de facto* method for rapidly screening cultured organisms [43].

SELDI, Surface-enhanced laser desorption ionization, is a variation of MALDI that binds proteins to a surface with a substrate, allowing interferences to be washed away. Though this ionization method is less frequently implemented than other strategies, discoveries made using SELDI have progressed into commercialized tests such as OVA1, which is an IVDMIA (in vitro diagnostic multivariate index assay) with clinical utility in directing exploratory surgery for women with abdominal masses [44].

Direct atmospheric ionization sources are relatively recent innovations that have promise in complementing traditional pathological examination of tissues. Conventionally, tissue to be examined is fixed and sectioned and subjected to a series of stains (dye-based or immunology- based) that when interpreted by a trained pathologist, can provide a diagnosis. The interpretation of a tissue section analyzed by mass spectrometry expands the interpretation by several dimensions. Rather than being limited to binding epitopes or dye affinity, the direct ionization of tissue provides molecular insight with a unique analytical specificity. The most commonly applied direct ionization techniques are Desorption Electrospray Ionization (DESI) [45] and Laser Ablation Electrospray Ionization (LAESI) [46], though other iterations are also being developed rapidly [47]. These methods have the possibility of providing unique insight into the molecular characterization of tissue by enabling discrete analysis of areas of differential pathology. The adoption of ionization techniques (in addition to MALDI) has enabled the analysis of biological surfaces and thus generated the field of “imaging mass spectrometry” [48–54].

### Fragmentation

Collision-induced dissociation (CID) is the most common method for the fragmentation of parent ions, and it is applied in triple quadrupole mass spectrometers and in hybrid instruments. It involves the application of an electrical potential to an ion into a region of high concentration collision gas (nitrogen or argon). It can provide different degrees of analytical specificity by adjusting the applied potential. Other mechanisms for fragmentation are compared to CID.

Higher-energy collisional dissociation (HCD) is a CID method applied specifically to the Orbitrap that involves a multipole collision cell that removes the low mass cutoff involved with traditional CID, enabling isobaric tag quantification.

Electron transfer dissociation (ETD) has some benefits compared to CID, which can be prohibitive in accurately assessing certain PTMs due to the energy that is required to be applied to the ions. ETD allows for more complete sequencing of modified peptides, and it may have a role in better characterizing phosphorylated and glycosylated peptides [55]. Due to its recent development and commercialization, ETD may provide access to the improved characterization of potential biomarkers compared to other collision strategies.

## Analytical challenges

### False discovery rate

Historically, biochemical analysis did not include analyzing >~1000 peptides or metabolites in a single experiment. A consequence of multiple comparisons is that “by chance”, analytes will prove to be statistically significant. Straightforward corrections to this are easy to implement but may be too stringent, typically normalizing the calculated significance directly to the number of comparisons made (e.g., Bonferroni correction [56, 57]). Contemporary methods are less stringent but involve more complicated calculations, and typically involve analyzing the distribution of significant features, such as with the determination of the q-value [58, 59]. Even with the abundance of technical challenges and innovations that will surround mass spectrometry in the decades to come, the omnipresent burden of false discovery will always need recognition when transitioning from biomarker discovery to biomarker validation. Key input will be required from practiced clinicians, biochemists, and clinical chemists prior to biomarker development for key insights into markers that have low likelihood of passing larger clinical trials due to dependence on other co-variates.

### Pre-analytical considerations

One issue with biomarker discovery is that even with the sophisticated software and analyzers available, if the samples have been affected by a factor not being tested for (a common drug in a disease state, or a different handling prior to analysis) there may be “false discoveries” not on the basis of disease, but by process. A seminal example of this is a study of ovarian cancer impacted by differential pre-analytical treatments [60, 61]. Other common scenarios involve patient cohorts compared to healthy cohorts where the patient cohorts are in an advanced stage of disease requiring medication. Some palliative drugs for treating cancer patients, such as steroids, have a gross impact on patient physiology so that even if patients have not yet been treated with chemotherapy, markers of disease could be identified that are simply a result of steroid use [62].

### Quantification

The statistics that are calculated to determine if a feature will be transitioned from a discovery stage to a pre-validation or validation stage rely on accurate and precise quantification. The quantification strategies for proteins and metabolites are typically quite different. Protein digests are typically quantified by spectral counting or by using isobaric tags (e.g. ITRAQ) [63, 64]. Metabolite quantification is typically performed by mixing an extract with known quantities of internal standards [65].

Isobaric tags are chemical derivatization agents that allow for quantification of proteins from different conditions (e.g., treated or non-treated). They work on the principle that after a protein is digested, the labels will produce products that are isobaric. After fragmentation in the mass spectrometer, however, they will provide both a peptide fingerprint (for identification) as well as a series of mass tags that enable relative quantification [66]. There are some caveats to isobaric tag quantification (in addition to those generally encountered during bottom-up analysis in general). Namely, there is a “ratio compression” effect that limits the magnitude of change observed when comparing states using tagging chemistry. Approaches have been suggested for mitigating these effects [67]. Other approaches (though not as directly applicable in mammalian organisms) include the metabolic incorporation of amino acids for quantitative comparison [68]. A recent study describes a method for isobaric tags for glycans titled QUANTITY that in addition to enabling quantification also enhances sensitivity [69].

Small molecule quantification has several more robust options for quantification compared to proteins, with the benefit that the molecule analyzed is typically intact, and not a digestion product. The most direct approach to metabolite quantification is isotope dilution, where an isotopically labeled analogue of a metabolite is mixed with an extract and the intensity ratio used to back-calculate the concentration of the extracted metabolite. An alternative to adding a metabolite prior to an extract prior to analysis is to add it after the column, (e.g., post-column infusion). This method had the benefit of providing, to an extent, relative quantification for an entire chromatographic analysis [70].

## Recent examples OF Biomarkers

### TMAO

Trimethylamine-N-oxide (TMAO) has been evaluated as a marker to predict major adverse cardiac events and other events. It also holds significance as a marker that not only relies on human pathophysiology, but also interaction with the metabolism of gut microbiota: microbiota metabolize phosphatidylcholine to TMAO, and the levels of this metabolite are associated with risk of death by myocardial infarction and stroke [71, 72].

#### Sarcosine

One of the very first proposed contemporary mass spectrometry-based metabolic biomarkers was sarcosine: proposed as a marker for aggressive prostate cancer. Sarcosine is an *n*-methyl derivative of glycine. Its biological mechanism and clinical applicability are still being actively evaluated [73, 74].

#### (*R*)-2-hydroxygularate

Coined the first “Oncometabolite,” (R)-2-hydroxygularate is one of the first metabolites ubiquitously produced at high levels due to a gain-in-function mutation in a gene in the TCA cycle in gliomas and acute myeloid leukemias [24–26, 75]. This marker may provide insight both into tumorigenesis as well as management of progression or treatment.

#### OVA1

OVA1 is an in vitro diagnostic multivariate index assay (IVDMIA) used for the management of women with pelvic masses that are suspected for ovarian cancer. Its clinical application is to help non-gynecological oncologists refer for surgery to determine whether a mass is cancerous. OVA1 is the first FDA-cleared IVDMIA, and it uses a combination of CA125 and 4 other protein markers to determine a score that assists a clinician in the assessment of the patient’s risk of ovarian cancer [44, 76].

#### National consortia supporting biomarker development and clinical proteomics

Over the past two decades, mass spectrometry has found a new home in helping improve the clinical management of disease. Consortia have developed, notably the National Cancer Institute (NCI) Clinical Proteomic Tumor Analysis Consortium (CPTAC) and the Early Detection Research Network (EDRN). These consortia have directly impacted our ability to discovery new biomarkers. The first 5 years of CPTAC focused on removing significant technical barriers in proteomic measurements and improved the accuracy, efficiency ad reproducibility in the identification and quantification of proteins. The second 5 years analyzed the tumor specimens from The Cancer Genome Atlas (TCGA) and produced proteomic data including PTMs in order to connect genomic alterations with proteomics. The EDRN has developed a number of new cancer biomarkers and translated them into clinical diagnostics. Five of these clinical diagnostics have received FDA clearance or approval. These clinical diagnostics will have significant impacts on the early detection and management of cancer. In parallel, mass spectrometry vendors have developed technologies that improve the quantitative aspects of analysis, providing the necessary accuracy and precision required for robust biomarker discovery. Technology is rapidly advancing to further improve analytical specificity. These technological improvements will have direct impacts on our ability to discovery new biomarkers, for example, the recent development of the ion funnel approach by Smith et al. [77].

## Developing a strategy for biomarker discovery

There are two major factors that will drive successful mass-spectrometry based biomarker discovery studies. The first will be implementing strict experimental design constraints that help insure the biomarkers that are discovered reflect pathophysiology and not analytical artifacts. It’s important to recognize that after surveying hundreds or thousands of features as is common in mass-spectrometry based analysis that false discoveries will be made. Utilization of contemporary approaches to false discovery rate correction such as the q-value correct the significance of findings based on the underlying distribution, and tend to overcorrect less than older methods such as Bonferroni correction. After ensuring strict experimental design, a decision needs to be made about the matrix to be examined. There are many options and the opinion generally depends on investigator preference. Options include examination of primary patient tissue, patient blood, patient urine, patient cerebrospinal fluid, cell culture, animal model, and others. While the ideal biomarker would be present in high concentrations in a patient blood, some investigations, particularly for tumor biomarkers, focus on protein or metabolic categorization of tumor tissue. This workflow works under the assumption that a protein or metabolite found highly enriched in tumor may be secreted into the blood. As biomarker studies as high throughput screens, they may lack the analytical sensitivity to detect the protein isoform or metabolite in blood without targeted enrichment or targeted mass spectrometry analysis. Moreover, tissue lends itself to direct analysis using new atmospheric ionization detection mechanisms, such as LAESI or DESI. Using these for mass spectrometry imaging experiment provide additional information in the way of spatial resolution, but may lack the sensitivity or precision of conventional protein mass spectrometry.

**Table 1:** Key technological developments for novel MS-based biomarker discovery

	Protein	PTM	Flux	Small molecule metabolite
Detection Schema	Shotgun, top-down proteomics	Shotgun, top-down proteomics	Kinetic flux profiling	Imaging-MS, LC–MS
Key Technology Developments	Dynamic range, throughput, quantification strategies	Analytical sensitivity, throughput, stability	Algorithms for relating labeling patterns and flux, complete understanding of pathways for constraining calculation of flux	Analytical sensitivity, structure deconvolution, isobaric separation
Possible Clinical Applications	Cancer and cardiovascular screening and prognosis	Cancer and cardiovascular screening and prognosis	Pharmacometabolomics	Basis for new scanning technologies, gut microbiome implications, screening of oncometabolites

Whether the specimens were tissue or liquid, the specimen could be analyzed for either proteins or metabolites. As mentioned previously, the emphasis of protein biomarker discovery is still on elucidating disease specific protein isoforms. To leverage contemporary technology, application of ETD as a fragmentation method improves the capacity to detect labile modifications compared to the historical collision-activated dissociation. Robust protein quantification generally requires isobaric tag labeling—for peptides this is generally accomplished using iTRAQ, however glycans could be quantified using QUANTITY. QUANTITY is the most recent method described for macromolecule detection and could provide insight into glycan modification.

Metabolite detection may be accomplished using a targeted approach by generating a library of MRMs associated with known standards or by untargeted analysis. Targeted analysis will typically leverage specific internal standards, and as a consequence will require consideration of what is commercially available as well as a priori hypothesis generation. Untargeted analysis has more difficult considerations regarding quantification, though using an internal standard infusion may help with both quantification as well as mass accuracy. A primary challenge with unknown small molecule metabolite biomarker discovery is elucidating the structure, especially considering the possibility of isobaric compounds.

With proper study design and utilization of cutting-edge enhancements (summarized in Table 1) to transitional mass-spectrometry biomarker discovery workflows, an abundance of potential clinical biomarkers should be generated. Those that will succeed will be vetted by chemists, biologists, and clinicians on the basis of mechanistic likelihood. Their transition to the clinic will be clearly defined by an intended use and only implemented after both analytical and pre-analytical requisites are clearly defined by performing laboratories.

## Conclusions

The greatest unmet clinical needs in biomarker discovery are those tests that provide early intervention when a patient would present otherwise healthy (e.g., cancer or cardiovascular disease) as well as those tests that aid clinical decision making with improved clinical outcomes.

The MS-based biomarker discovery field has been split into camps of pessimism and cautious optimism. The field has matured considerably with regards to emphasis on good experimental design and the need to reduce false discovery. These lessons should certainly be considered as MS-based discovery space enters brand new realms of analysis (e.g., intra-operative margin detection, metabolomics, metabolic flux profiling, and MS-based imaging). Diamandis has recently suggested the creation of a “rare” tumor marker repository of proteolytic peptides [78]. This recommendation, though with limitations, aligns with an NIH/NCI initiative for personalized medicine. The feasibility of this initiative will be limited by the relative cost and diagnostic accuracy of an MS-based approach compared to a nucleic acid sequencing approach. However, the limited success of shotgun proteomics in the development of clinical biomarkers should not dissuade aspiring clinical chemists or other scientists involved in biomarker discovery from adopting mass spectrometry technology as a biomarker discovery tool. Early signs of success have been evident in fields of cardiovascular risk and cancer using MS technology with a different analytical paradigm: small molecules instead of shotgun proteomics. A much-deserved respite from the over-pessimism in the field could be provided by focusing on the successes of contemporary MS applications in the clinic. Expectations for the ideal MS-based biomarker should reflect the significant recent and future improvements in its technological basis.
